# Oseltamivir Prescribing in Pharmacy-Benefits Database, United States, 2004–2005^1^

**DOI:** 10.3201/eid1408.080074

**Published:** 2008-08

**Authors:** Justin R. Ortiz, Laurie Kamimoto, Ronald E. Aubert, Jianying Yao, David K. Shay, Joseph S. Bresee, Robert S. Epstein

**Affiliations:** * Centers for Disease Control and Prevention, Atlanta, GA, USA; † Medco Health Solutions, Inc., Franklin Lakes, New Jersey, USA; 2 Current affiliation: University of Washington, Seattle, WA, USA.

**Keywords:** Human influenza, influenza A virus, avian influenza, influenza A drug therapy, influenza A virus subtype H5N1, dispatch

## Abstract

We reviewed information from a US pharmacy benefits manager database from 2004 through 2005 during periods with little influenza activity. We calculated rates of oseltamivir prescriptions to enrollees. Prescription rates increased significantly from 27.3/100,000 in 2004 to 134/100,000 in 2005 (p<0.05), which suggested that personal stockpiling of oseltamivir occurred.

From 2003 through 2006, avian influenza virus (H5N1) spread from Southeast Asia to Africa, Europe, and the Middle East (*1*), and media coverage about the risk for human infection and the potential for an influenza pandemic increased. Two classes of medications are available to treat influenza: neuraminidase inhibitors (NIs), which include oseltamivir and zanamivir, and adamantanes, which include amantadine and rimantadine (*2*). NIs are recommended by the World Health Organization for treatment of avian influenza virus (H5N1) infection because isolates have demonstrated adamantane resistance (*3*). During the fall of 2005, NIs were in limited supply (*4*).

In 2005, concern was expressed in the medical literature about possible personal stockpiling of NIs for use during an influenza pandemic (*5*). We undertook this study to look for evidence of oseltamivir stockpiling, to understand the magnitude of the practice, and to discern who was receiving and prescribing these drugs. We collaborated with a pharmacy benefits management company to examine antiviral prescriptions and oseltamivir prescription filling in the United States during calendar weeks 36–44 in 2004 and 2005. These weeks were chosen because they had little influenza activity in either year and because reports of oseltamivir stockpiling occurred during this period in 2005 (*6*–*9*).

## The Study

We used a database from Medco Health Solutions, Inc. (Franklin Lakes, NJ, USA), a pharmacy benefits management company serving >50 million US members. We examined filled prescriptions for oseltamivir by members from January 2002 through May 2006. Available member data included demographic information, medication dispensed, prescriber identification, and pharmacy dispensing history. Member-level historic pharmacy dispensing data were used to assign members into chronic disease classifications (*10*). Prescribers were cross-referenced with an American Medical Association member database to determine specialty and years since medical school graduation. We were able to cross-reference 64% of prescribing physicians by specialty and years since medical school graduation. The Centers for Disease Control and Prevention (Atlanta, GA, USA) determined that institutional review board approval was not needed for this study because we received aggregated data that was anonymous and not identified.

To assess media coverage, we queried the LexisNexis US News database (www.lexisnexis.com) for total weekly news reports from August 1, 2003, through August 30, 2006, referring to avian influenza and oseltamivir. Weekly virologic data from the World Health Organization and National Respiratory and Enteric Virus Surveillance System collaborating laboratories were used to assess US influenza activity during 2004 and 2005 (*6*,*7*).

Oseltamivir prescription rates were calculated per 100,000 enrolled members and per 1,000 prescribing physicians. Binomial distributions were used to estimate variances for rates. Relative rate ratios (RRs) and 95% confidence intervals (CIs) were calculated for 2004 and 2005 data. P values <0.05 were considered statistically significant. Analyses were performed with SAS version 9.0 statistical software (SAS Institute, Cary, NC, USA).

**Figure 1:**
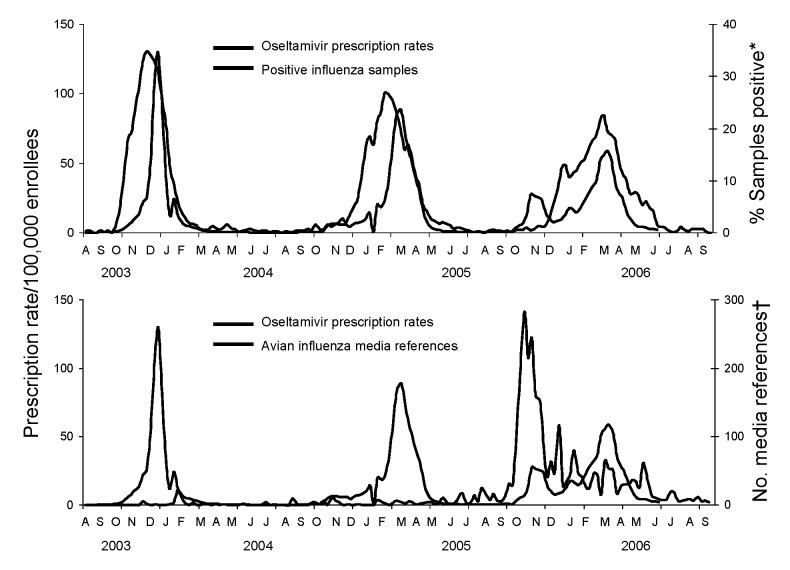
Weekly influenza activity, oseltamivir prescription rates for enrollees of all ages, and LexisNexis references to avian influenza and oseltamivir, United States, 2003–2006. *World Health Organization and National Respiratory and Enteric Virus Surveillance System collaborating laboratories in the United States. †LexisNexis US News database query for weekly news reports referring to “(avian or bird or H5N1) and (flu or influenza) and (Tamiflu or oseltamivir).”

Weekly rates of filled prescriptions for oseltamivir and percentage of samples positive for influenza from October 1, 2002, through June 1, 2006, were temporally associated before the 2005–06 influenza season (Figure 1). During the fall of 2005, prescriptions for oseltamivir increased without an associated increase in the percentage of samples testing positive for influenza. In contrast, during the same period, there was a temporal relationship between weekly oseltamivir prescription rates and media reports of avian influenza and oseltamivir (Figure 1).

The proportion of oseltamivir prescriptions to total anti-influenza prescriptions increased from 37.0% in 2004 to 76.9% in 2005 (Table 1). The 2005 oseltamivir prescription rate of 133/100,000 during weeks 36–44 was ≈5× the 2004 rate of 27.3/100,000 (RR 4.88, 95% CI 4.79–4.97) (Table 1).

**Table 1:** Anti-influenza drug prescription rates/100,000 enrollees and proportions of all anti-influenza drug prescriptions, United States, weeks 36–44, 2004 and 2005*

Medication	Anti-influenza prescription rates/100,000 enrollees of all ages, weeks 36–44					% Total anti-influenza prescriptions	
	2004	2005	Rate ratio (2005/2004)	95% Confidence interval		2004	2005
Neuraminidase inhibitors							
Oseltamivir	27.3	133	4.88	4.79–4.97		36.99	76.89
Zanamivir	0.35	1.39	4.00	3.38–4.75		0.47	0.80
Adamantanes							
Amantadine	41.7	36.3	0.87	0.85–0.89		56.53	20.93
Rimantadine	4.43	2.37	0.54	0.50–0.58		6.00	1.37
All anti-influenza drugs	80.0	173.2	2.17	2.14–2.19		100	100

*Sums do not equal 100% because of rounding.

Women were more likely to receive oseltamivir prescriptions than men in 2004 (RR 1.19, 95% CI 1.16–1.24) and 2005 (RR 1.07, 95% CI 1.05–1.09). Prescription rates increased from 2004 to 2005 for all age groups (Table 2). The highest prescription rates in 2005 were for persons 50–64 years of age (211/100,000) and those >65 years of age (168/100,000). Members <18 years of age had a >7-fold increase in prescription rates from 2004 to 2005.

**Table 2:** Oseltamivir prescription rates/100,000 enrollees by age and chronic disease classification, United States, weeks 36–44, 2004 and 2005

Characteristic	2004	2005	Rate ratio (2005/2004)	95% Confidence interval
Age group, y				
<1	0.26	10.65	40.7	(5.6–294)
1–4	8.95	65.95	7.4	(6.3–8.6)
5–17	11.12	81.28	7.3	(6.8–7.9)
18–24	19.55	81.42	4.2	(3.8–4.5)
25–49	33.08	120.97	3.7	(3.5–3.8)
50–64	63.33	211.26	3.3	(3.2–3.4)
>65	69.04	168.20	2.4	(2.4–2.5)
Chronic disease classification*				
Pulmonary	77.66	268.50	3.46	(3.33–3.60)
Immune deficient	67.75	240.16	3.54	(3.21–3.91)
Neurologic	54.57	207.43	3.80	(3.47–4.15)
Cardiac	54.11	196.92	3.64	(3.54–3.74)
Diabetes	50.85	143.85	2.83	(2.64–3.02)
Chronic disease absent	14.07	89.50	6.36	(6.10–6.62)
*Rates from chronic disease classification include only enrollees >18 y of age. Except for the chronic disease absent category, all chronic disease classifications were not mutually exclusive. Chronic disease classification is derived from the chronic disease index, which has been validated (*10*).				

Among adults, prescription rates were consistently higher in 2005 than in 2004, irrespective of chronic disease classification (Table 2). In 2005, the highest oseltamivir prescription rate was 268.5/100,000 for enrollees with pulmonary disease, and the lowest rate was 89.5/100,000 for those without chronic disease. The greatest rate increase from 2004 to 2005 occurred among those without chronic disease (RR 6.36, 95% CI 6.10–6.62). Among enrollees of all ages during weeks 36–44 in 2005, approximately one third of oseltamivir prescriptions were filled by members without chronic disease.

**Figure 2:**
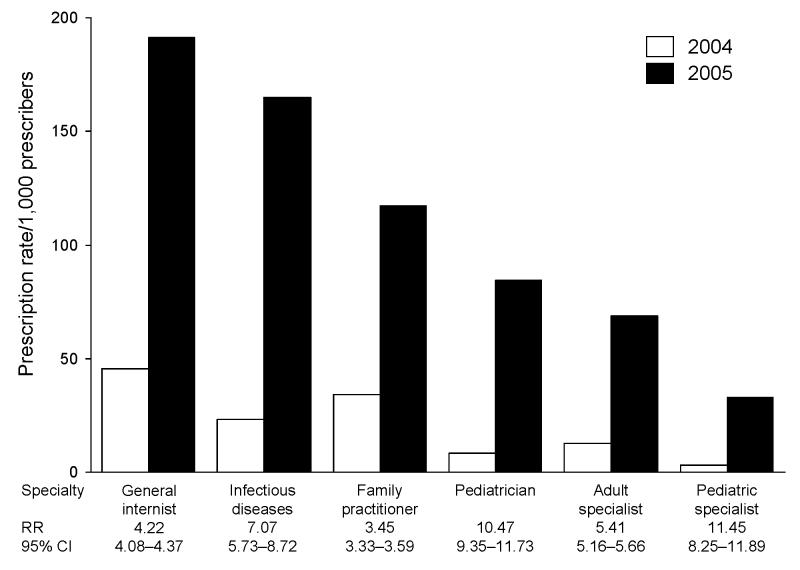
Oseltamivir prescription rates/1,000 prescribers by specialty, United States, weeks 36–44, 2004 and 2005. Infectious diseases classification includes pediatric and adult infectious diseases specialists. All classifications are mutually exclusive. RR, rate ratio; CI, confidence interval.

In 2004 and 2005, general internists had the highest average prescribing rate for oseltamivir (45.3/1,000 and 191.1/1,000, respectively) (Figure 2). Pediatricians had the lowest rates in both years (2.9/1,000 and 32.7/1,000).

For all prescribers in 2004 and 2005, oseltamivir prescription rates increased with years since the prescriber’s medical school graduation. The lowest prescription rate in 2005 (1.7/1,000) was observed in prescribers who graduated from medical school in the previous 5 years, followed by prescribers with 5–10 years (4.9/1,000) and 11–19 years (6.6/1,000) since graduation. The highest rate (10.4/1,000) was observed in prescribers with >20 years since graduation.

## Conclusions

Rates of filled oseltamivir prescriptions during calendar weeks 36–44 increased from 2004 to 2005. These weeks in 2005 were noteworthy for increased media references to oseltamivir and avian influenza, although there was little influenza activity. Low levels of influenza-like illness and respiratory syncytial virus activity also were documented during this period (*6*,*11*).

Among Medco enrollees, the highest prescription rates were for groups with the greatest risk for influenza-associated complications: persons >50 years of age and adults with chronic diseases (*2*). However, oseltamivir prescription rates increased from 2004 through 2005 for each age and chronic disease category. Although absolute prescription rates were lower in persons <50 years of age, persons without chronic medical diagnoses, and children, these groups had higher rate increases from 2004 to 2005, suggesting that they and their caretakers were influenced to stockpile oseltamivir in 2005.

Physician prescribing rates for all specialties increased from 2004 to 2005. Prescription rates in 2004 were highest for general internists and family practitioners and likely reflect the primary care physician’s gatekeeper role as the entry point for those seeking medical care. Given the nature of their specialty, infectious diseases physicians may be asked to prescribe oseltamivir for personal stockpiles more than the typical provider. Because physicians need not honor all prescription requests, these increases may not be fully explained by increased patient requests because physician attitudes regarding personal stockpiling likely affected whether requests were made or honored.

Our study is subject to limitations. Although we studied a large, national population, our study population may not be nationally representative. In addition, our analyses were limited to prescriptions of oseltamivir that were filled by a pharmacy, and we do not know whether prescriptions were written with the expressed purpose of personal stockpiling for use during a pandemic.

In summary, our findings suggest that increased media reports during the fall of 2005 about the influenza (H5N1) epizootic prompted concern about the possibility of an influenza pandemic. This heightened concern led to an increase in filled oseltamivir prescriptions for personal stockpiling among a national pharmacy benefits member population. Subsequently, as influenza virus began circulating in early 2006, oseltamivir prescriptions corresponded more closely with virus activity. Efforts by federal and state governments to procure sufficient supplies of NIs to treat every patient likely to become ill during the next pandemic may quell demand in personal stockpiles. Education campaigns about appropriate use of antiviral medications that target physicians and patients during seasonal epidemics and pandemics may reduce inappropriate requests for oseltamivir and other drugs.
